# A Double-Blinded, Randomized, Placebo-Controlled Trial to Evaluate Efficacy, Safety, and Tolerability of Single Doses of Tirasemtiv in Patients with Acetylcholine Receptor-Binding Antibody-Positive Myasthenia Gravis

**DOI:** 10.1007/s13311-015-0345-y

**Published:** 2015-03-06

**Authors:** Donald B. Sanders, Jeffrey Rosenfeld, Mazen M. Dimachkie, Lisa Meng, Fady I. Malik, for the Tirasemtiv in Myasthenia Gravis Study Group

**Affiliations:** 1 Duke University Medical Center, Durham, NC 27710 USA; 2 University of California San Francisco, Fresno, CA USA; 3 University of Kansas Medical Center, Kansas City, KS USA; 4 Cytokinetics, Inc., San Francisco, CA USA

**Keywords:** Myasthenia gravis, tirasemtiv, CK-2017357, fast skeletal troponin activator

## Abstract

Tirasemtiv is a fast skeletal troponin activator that sensitizes the sarcomere to calcium and increases muscle force following subtetanic nerve input. In an animal model of myasthenia gravis (MG), single oral doses of tirasemtiv improved muscle force and reduced fatigability. The purpose of this study was to determine the effect of single doses of tirasemtiv on skeletal muscle function and fatigability in patients with generalized MG. Thirty-two patients with acetylcholine receptor-antibody positive MG and muscle weakness received single doses of tirasemtiv (250 mg or 500 mg) or placebo in a double-blind, randomized treatment sequence with each treatment separated by at least 1 week. Outcome measures included the Quantitative MG Score (QMG), MG Composite, Manual Muscle Testing, and forced vital capacity. At 6 h after dosing, tirasemtiv produced dose-related improvements from baseline in the QMG score (slope: –0.49 QMG point per 250 mg; *p* = 0.02) and in percent predicted forced vital capacity (slope: 2.2 % per 250 mg; *p* = 0.04). QMG improved >3 points in twice as many patients after 500 mg tirasemtiv than after placebo. Both doses of tirasemtiv were well tolerated; there were no premature terminations or serious adverse events. The results of this study suggest that tirasemtiv may improve muscle function in MG and will be used to support further development of tirasemtiv in neuromuscular diseases.

**Electronic supplementary material:**

The online version of this article (doi:10.1007/s13311-015-0345-y) contains supplementary material, which is available to authorized users.

## Introduction

In myasthenia gravis (MG), weakness and fatigue result from failure of signal transmission at the neuromuscular junction due to antibody binding of the acetylcholine receptor resulting in limited force production [1]. Treatment consists of acetylcholinesterase inhibitors and immunosuppression, although weakness and fatigue are still common in these patients [2].

Tirasemtiv (formerly CK-2017357) is an investigational drug that is a highly selective activator of the fast skeletal muscle troponin complex. It was developed as a means to increase muscle strength by amplifying the response of muscle when neuromuscular input is diminished in diseases such as MG that result in a decrease in muscle fiber action potentials and, consequently, a decrease in the number of muscle fibers contracting, thus leading to a decrease in muscle force production [3]. Tirasemtiv selectively activates the fast skeletal muscle troponin complex by increasing its sensitivity to calcium, thereby increasing skeletal muscle force in response to neuronal input and delaying the onset and reducing the degree of muscle fatigue [3]. Tirasemtiv slows the rate of calcium release from fast skeletal troponin, thus increasing its affinity for calcium and sensitizing muscle to calcium. As a consequence, the force–calcium relationship of fast skeletal muscle fibers shifts leftward and muscle force increases relative to control at submaximal activation. In preclinical models of nerve–muscle function and in humans, tirasemtiv amplified the response of muscle to submaximal nerve stimulation but not to tetanic stimulation [3, 4]; muscle force increased within the operating range of motor unit discharge rates associated with the voluntary activation of skeletal muscle. Increases in skeletal muscle strength and endurance have been observed after single doses of tirasemtiv in patients with amyotrophic lateral sclerosis (ALS) and patients with peripheral vascular disease and claudication [5, 6]. Pertinent to the current study, tirasemtiv decreased muscle fatigability, increased muscle force, and increased grip strength in a passive transfer rat model of MG [3].

The primary objective of this early-stage clinical study was to demonstrate an effect of single doses of tirasemtiv on measures of skeletal muscle function and fatigability in patients with generalized MG and persistent muscle weakness. Accordingly, in this hypothesis-generating phase 2 study, multiple assessments of skeletal muscle function and fatigability were made. The secondary objectives of the study were to evaluate and characterize the relationship, if any, between the doses and plasma concentrations of tirasemtiv and its pharmacodynamic effects; and to evaluate the safety and tolerability of tirasemtiv administered as single doses to patients with MG.

## Methods

This phase 2, double-blind, placebo-controlled, randomized study examined 2 single doses of tirasemtiv, 250 mg and 500 mg, and placebo in a 3-way crossover fashion. The study also measured the pharmacodynamic properties of tirasemtiv in patients with generalized MG on standard therapy. The study was conducted between 29 December 2010 (first patient screened) and 10 October 2012 (last patient visit completed) at 15 study centers in the USA. Independent ethics committees at each study site approved the protocol, and all patients provided written informed consent before the initiation of study-specific procedures. The study was conducted in compliance with the Declaration of Helsinki and registered under ClinicalTrials.gov identifier NCT01268280.

### Study Design

Patients were randomly assigned to 1 of 6 different treatment sequences. Each treatment sequence consisted of 3 dosing periods in which patients received single oral doses of 250 mg or 500 mg tirasemtiv or placebo, with a washout period of at least 7 days (up to a maximum of 10 days) between the individual doses. Treatment sequences for the 3 dosing periods were assigned as follows: sequence 1—placebo–tirasemtiv 250 mg–tirasemtiv 500 mg; sequence 2—placebo– tirasemtiv 500 mg–tirasemtiv 250 mg; sequence 3—tirasemtiv 250 mg–placebo–tirasemtiv 500 mg; sequence 4—tirasemtiv 250 mg–tirasemtiv 500 mg–placebo; sequence 5—tirasemtiv 500 mg–placebo–tirasemtiv 250 mg; and sequence 6—tirasemtiv 500 mg–tirasemtiv 250 mg–placebo. The single doses of tirasemtiv selected for this study (250 mg and 500 mg) were expected to produce mean plasma concentrations that increased the response of muscle to neuromuscular input, and were well tolerated by healthy male volunteers in a previous study [4].

Only specifically designated study site pharmacy staff were unblinded to treatment; the investigator, patient, and remaining study site clinical staff were blinded as to treatment assignment. An interactive web response system provided patient randomization assignment to the unblinded pharmacists who dispensed the study medications. All patients received 4 capsules during each dosing period. Depending on the assigned treatment sequence, each patient received either 4 capsules of tirasemtiv (125 mg each), 2 capsules containing tirasemtiv and 2 matching placebo capsules, or 4 matching capsules all containing placebo. All doses were preceded by an overnight fast (i.e., at least 8 h) from food (not including water) and followed by a fast from food (not including water) for 1 h postdose.

### Patients

Eligible patients were aged 18–80 years, had a body mass index between 18.0 and 36.0 kg/m^2^, and had an established diagnosis of MG. The diagnosis of MG was defined as clinical evidence of muscle weakness that could not be better explained by another disease and a history of an elevated acetylcholine receptor-binding antibody titer. Patients were Myasthenia Gravis Foundation of America clinical classification II or III, and had to have stable weakness and treatment for 4 weeks prior to randomization. Patients had to be able to perform all elements of the Quantitative Myasthenia Gravis (QMG) assessment [7], with QMG scores of 2 or 3 in ≥2 of the following muscle groups: right or left arm outstretched, head lift, and right or left leg lift at 45^o^. Patients could not have received cholinesterase inhibitors for 12 h before each dose. Key exclusion criteria included history of any chronic degenerative, psychiatric, or neurologic disorder other than MG that produced weakness or fatigue; other major chronic or debilitating illnesses in the 6 months prior to study entry; renal insufficiency (serum creatinine >2.5 mg/dl or receiving dialysis); other myasthenic syndromes; receipt of intravenous immunoglobulin, plasmapheresis treatment, or changes to immunosuppressive treatments in the 6 weeks prior to the first dose of study drug; receipt of rituximab in the 3 months prior to study entry, receipt of any investigational drug or device in the 30 days prior to first dose of study drug; or any prior treatment with tirasemtiv.

### Outcome Measures

Safety assessments included physical examinations, electrocardiograms, vital signs, and clinical laboratory evaluations performed at screening and at specified times during the study. All adverse events, whether volunteered, elicited, or noted on physical examination or through laboratory evaluations, were recorded. Pharmacodynamic assessments included the following: QMG score [7], MG Composite (MGC) score [8], the percent predicted forced vital capacity (FVC), and manual muscle testing (MG-MMT) of selected muscles [9]. The QMG score is a 39-point scale that assesses diplopia (to primary and lateral gaze) right or left, ptosis, facial muscles, swallowing, dysarthria with counting, right and left arm held outstretched at 90^o^, FVC (percent predicted), right and left hand grip, head lift 45^o^ supine, and right and left leg held outstretched at 45^o^ supine [7]. Each assessment is graded from 0 to 3 points, with 3 points representing the most severe dysfunction; thus, lower scores represent better function. The MGC is a validated, 10-item assessment based on physician examination and patient history, in which a 3-point improvement on a total scale of 0–50 indicates clinical improvement [8]. The MG-MMT assesses neck flexion and extension, shoulder abduction (deltoid), and hip flexion [9]. Pharmacodynamic outcomes were assessed at screening (QMG only) and at 0, 3, 6, and 24 h (QMG, FVC, MGC, MG-MMT) after dosing.

### Statistical Considerations

A sample size of 6 patients per treatment sequence (total of 36 patients) was estimated to provide 90 % power to detect a difference in mean QMG score of 0.60 SDs with a 2-sided significance level of 0.05. Descriptive statistics are presented for continuous (n, mean, median, SD, range) and categorical (number and percentage of patients) variables. Adverse events were categorized using the Medical Dictionary for Regulatory Activity version 10.1. Statistical analyses were performed using SAS for Windows, Version 9.2 (SAS Institute, Cary, NC, USA).

Change from predose baseline in QMG total score, MGC total score, and FVC were analyzed by time using repeated measures analysis of covariance analyses with treatment, sequence, and period as fixed effects, the corresponding predose baseline value from each period as the covariate, and patient as a random effect in the model. Treatment effect over both 3 and 6 h postdose was analyzed by including a time point variable in the model statement. For FVC, with 3 trials performed at each time point, the changes from predose baseline were analyzed by time using repeated measures analysis of covariance analyses with treatment, sequence, trial, and period as fixed effects, the corresponding baseline value as a covariate, and patient as a random effect in the model. *p*-Values and 95 % confidence intervals were calculated for treatment differences at each time point between active dose levels and placebo. No primary efficacy variable was prespecified and no adjustments were made for multiple comparisons.

The safety population (all patients who received any amount of study drug) was used for the evaluation of patient disposition, demographics, clinical characteristics, and all safety outcomes. The pharmacodynamic population (all patients in the safety population who had at least 1 postbaseline pharmacodynamic assessment and no major protocol deviations) was used to evaluate pharmacodynamic outcomes.

## Results

### Patients

A total of 32 patients (of 36 planned) were enrolled in the study and randomly assigned to 1 of 6 treatment sequences of tirasemtiv (250 mg and 500 mg) and placebo. The mean age of the patients was 54.4 years; half were male; and the study population was predominantly white (66 %) (Table 1). All patients were included in the safety population, and all patients were eligible to be included in the pharmacodynamics population.

**Table 1:** Demographics and clinical characteristics at baseline

	All patients (*n* = 32)
Mean age, years (SD)	54.4 (19.8)
Age range (years)	19.0–85.0
Ethnicity, *n* (%)	
White	21 (65.6)
Asian	2 (6.3)
Black/African American	3 (9.4)
Other	6 (18.8)
BMI, mean kg/m^2^ (SD)	29.2 (4.7)
QMG total score, mean (SD)	16.6 (4.9)
MGC score, mean (SD)	11.0 (6.6)
FVC, mean l/min (SD)	2.98 (0.89)
FVC % predicted, mean (SD)	77.4 (16.2)
MG-MMT total score, mean (SD)	6.3 (4.5)

BMI = body mass index; QMG = Quantitative Myasthenia Gravis; MGC = Myasthenia Gravis Composite; FVC = forced vital capacity; MMT = manual muscle tests

### Pharmacodynamic Outcomes

**Fig. 1:**
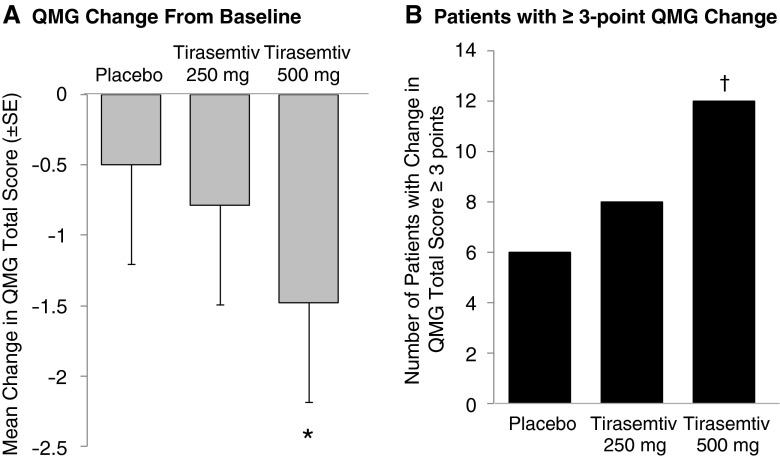
Quantitative Myasthenia Gravis (QMG) total scores and patients with change in QMG total score ≥3 points at 6 h after dosing. The (A) least square mean change in QMG total score by treatment and (B) number of patients who achieved a ≥3-point change in QMG total score (indicating clinical improvement) are shown. Error bars represent SE. *p = 0.02 *vs* placebo; ^†^ *p* = 0.098 *vs* placebo based on χ^2^ test

No statistically significant differences in QMG total score between placebo and either tirasemtiv dose were noted at 3 h after dosing (Table 2). At 6 h after dosing, dose-related improvement from baseline (i.e., decreases) in the QMG total score were found with an increase of –0.49 QMG points per 250 mg (*p* = 0.02) and in percent predicted FVC (slope: 2.2 % per 250 mg; *p* = 0.04). The least squares mean differences (SE) between treatment and placebo were –0.3 (0.4) for tirasemtiv 250 mg (*p* = 0.48) and –1.0 (0.4) for tirasemtiv 500 mg (*p* = 0.02), as shown in Fig. 1A. In a responder analysis, twice as many patients receiving tirasemtiv 500 mg improved by ≥3 points in their QMG total score compared with those receiving placebo (*p* = 0.098) (Fig. 1B).

**Table 2:** Quantitative myasthenia gravis (QMG) total score change (pharmacodynamic population)

	Placebo (*n* = 32)	Tirasemtiv 250 mg (*n* = 32)	Tirasemtiv 500 mg (*n* = 32)
Baseline QMG, mean total score (SD)	15.8 (5.3)	15.5 (5.1)	15.8 (5.0)
QMG change from baseline to hour 3, differences in LSM from placebo (95 % CI)	NA	0.1 (–1.0 to 1.3)	–0.2 (–1.3 to 0.9)
QMG change from baseline to hour 6, differences in LSM from placebo (95 % CI)	NA	–0.3 (–1.1 to 0.5)	–1.0 (–1.8 to –0.2)*

LSM = least square means; CI = confidence interval; NA = not applicable

*p < 0.05

FVC (percent predicted) at either dose of tirasemtiv was not significantly different from placebo at 3 h, and no statistically significant dose trend was observed (Table 3). At 6 h, however, compared with placebo, the least square mean (SE) change from baseline in percent predicted FVC was 7.0 % (2.1 %) for tirasemtiv 250 mg (*p* < 0.01) and 4.5 % (2.1 %) for tirasemtiv 500 mg (*p* = 0.03).

**Table 3:** Forced vital capacity (FVC) and percent predicted FVC (pharmacodynamic population)

	Placebo (*n* = 32)	Tirasemtiv 250 mg (*n* = 32)	Tirasemtiv 500 mg (*n* = 32)
Baseline FVC, mean l/min (SD)	3.0 (0.8)	3.0 (0.9)	3.1 (0.9)
FVC change from baseline to hour 3, differences in LSM from placebo (95 % CI)		0.04 (–0.02 to 0.10)	0.03 (–0.03 to 0.09)
FVC change from baseline to hour 6, differences in LSM from placebo (95 % CI)		0.17 (0.05, 0.29)*	0.06 (–0.07 to 0.18)
Baseline percent predicted FVC, mean % (SD)	80.9 (20.5)	81.5 (17.8)	81.8 (20.2)
Percent predicted FVC change from baseline to hour 3, differences in LSM from placebo (95 % CI)		–0.31 (–2.51 to 1.88)	–1.95 (–4.14 to 0.24)
Percent predicted FVC change from baseline to hour 6, differences in LSM from placebo (95 % CI)		6.98 (2.79–11.17)*	4.53 (0.34–8.73)^†^

LSM = least square means; CI = confidence interval

*p < 0.01; ^†^ *p* < 0.05

Overall, tirasemtiv did not show evidence of a positive effect on the MGC and MG-MMT scores (data not shown).

### Safety

No patients experienced a serious adverse event during the study, and no patients discontinued from the study because of an adverse event. Overall, 25 (78.1 %) patients had at least 1 treatment-emergent adverse event (Table 4). The most commonly reported treatment-emergent adverse events were dizziness (46.9 % on tirasemtiv and 6.3 % on placebo) and headache (12.5 % on tirasemtiv and 9.4 % on placebo). The duration of dizziness episodes ranged from 0.3 to 73.0 h. Most adverse events were mild or moderate in severity; 1 patient reported flushing as an adverse event of severe intensity with tirasemtiv 500 mg.

**Table 4:** Summary of safety (safety population)

	Placebo (*n* = 32)	Tirasemtiv 250 mg (*n* = 32)	Tirasemtiv 500 mg (*n* = 32)
Patients with ≥1 treatment-emergent adverse event	13 (40.6)	12 (37.5)	21 (65.6)
Patients with a severe adverse event	0	0	1 (3.1)
Adverse events occurring in ≥2 patients			
Dizziness	2 (6.3)	7 (21.9)	13 (40.6)
Headache	3 (9.4)	2 (6.3)	2 (6.3)
Dyspnea	0	1 (3.1)	2 (6.3)
Pollakiuria	1 (3.1)	0	3 (9.4)
Balance disorder	0	0	3 (9.4)
Feeling drunk	0	2 (6.3)	1 (3.1)
Anxiety	0	1 (3.1)	1 (3.1)
Blurred vision	0	1 (3.1)	2 (6.3)
Diarrhea	2 (6.3)	0	2 (6.3)
Dry mouth	1 (3.1)	0	1 (3.1)
Vomiting	1 (3.1)	0	1 (3.1)
Urinary tract infection	0	1 (3.1)	1 (3.1)
Muscle spasm	0	1 (3.1)	2 (6.3)

Values are given as n (%)

## Discussion

The results from this study suggest that tirasemtiv was well tolerated and may improve function in patients with MG. Dose-related improvements in QMG were observed with tirasemtiv, and twice as many patients had clinically significant improvements in QMG (>3 points) at 6 h after the 500 mg dose compared with placebo. Adverse events reported in this study were consistent with those observed in previous studies conducted in healthy volunteers [4], patients with ALS [5], and patients with calf claudication [6], most notably dizziness (which was dose-dependent), ataxia, and nausea. Adverse events were mostly mild in severity.

Patients did not receive their usual dose of pyridostigmine prior to the baseline assessment in order to ensure that there was some dysfunction at baseline. It is possible that the modest treatment effects observed actually underestimate the potential effect of tirasemtiv if it were to be given with pyridostigmine, as their mechanisms should be complementary; the possibility that clinical benefit would be greater in patients taking pyridostigmine warrants further evaluation.

A limitation of this study was the small sample size, which was not sufficient to prove clinical efficacy. As no primary efficacy end point was prespecified, there was no adjustment of *p*-values for multiple comparisons; thus, our findings should be considered as hypothesis-generating. Additionally, the short duration of the study does not provide information on long-term effects of treatment with tirasemtiv, which will be important for chronic diseases such as ALS and MG. Patients with more severe disease, such as Myasthenia Gravis Foundation of America grade IV, and patients without acetylcholine receptor-binding antibodies were excluded from the study, and the results may not be generalizable to patients with those disease characteristics. Tirasemtiv provides only functional improvement, and is not likely to reverse the immunologic processes that drive the pathogenesis of MG or prevent disease progression.

In conclusion, this study indicated that tirasemtiv may improve muscle weakness and fatigue associated with MG. The results from this study support further development of tirasemtiv to investigate its potential as a treatment for MG.

## Electronic supplementary material

Below is the link to the electronic supplementary material.

ESM 1 (PDF 507 kb)

ESM 2 (PDF 19 kb)

## References

[1] Drachman DB (1994). Myasthenia gravis. N Engl J Med.

[2] Conti-Fine BM, Milani M, Kaminski HJ (2006). Myasthenia gravis: past, present, and future. J Clin Invest.

[3] Russell AJ, Hartman JJ, Hinken AC (2012). Activation of fast skeletal muscle troponin as a potential therapeutic approach for treating neuromuscular diseases. Nat Med.

[4] Hansen R, Saikali KG, Chou W (2014). Tirasemtiv amplifies skeletal muscle response to nerve activation in humans. Muscle Nerve.

[5] Shefner J, Cedarbaum JM, Cudkowicz ME (2012). Safety, tolerability and pharmacodynamics of a skeletal muscle activator in amyotrophic lateral sclerosis. Amyotroph Lateral Scler.

[6] Bauer TA, Wolff AA, Hirsch AT (2014). Effect of tirasemtiv, a selective activator of the fast skeletal muscle troponin complex, in patients with peripheral artery disease. Vasc Med.

[7] Barohn RJ, McIntire D, Herbelin L, Wolfe GI, Nations S, Bryan WW (1998). Reliability testing of the quantitative myasthenia gravis score. Ann N Y Acad Sci.

[8] Burns TM, Conaway M, Sanders DB, MG Composite and MG-QOL15 Study Group (2010). The MG Composite: A valid and reliable outcome measure for myasthenia gravis. Neurology.

[9] Sanders DB, Tucker-Lipscomb B, Massey JM (2003). A simple manual muscle test for myasthenia gravis: validation and comparison with the QMG score. Ann N Y Acad Sci.

