# Supplementary material for: A Double-Blinded, Randomized, Placebo-Controlled Trial to Evaluate Efficacy, Safety, and Tolerability of Single Doses of Tirasemtiv in Patients with Acetylcholine Receptor-Binding Antibody-Positive Myasthenia Gravis
Source: Neurotherapeutics. 2015 Mar 6;12(2):455–60. doi: 10.1007/s13311-015-0345-y (PMC4404445; doi:10.1007/s13311-015-0345-y)
Supplement: Supplementary file 2 — (PDF 19 kb) [file 13311_2015_345_MOESM2_ESM.pdf]

A Double-Blinded, Randomized, Placebo-Controlled Trial to Evaluate Efficacy, Safety, and Tolerability of Single Doses of Tirasemtiv in Patients with Acetylcholinesterase Receptor-Binding Antibody Positive Myasthenia Gravis

SUPPLEMENTAL MATERIALS

The Tirasemtiv in Myasthenia Gravis Study Group:

- Jinsy Andrews, Hospital for Special Care, New Britain, CT
- Richard Barohn, University of Kansas Medical Center, Kansas City, KS
- Andrea Corse, Johns Hopkins University, Baltimore, MD
- Anahita Deboo, Drexel University College of Medicine, Philadelphia, PA
- Mazen M. Dimachkie, University of Kansas Medical Center, Kansas City, KS
- Kevin Felice, Hospital for Special Care, New Britain, CT
- Yadollah Harati, Baylor College of Medicine, Houston, TX
- Terry Heiman-Patterson, Drexel University College of Medicine, Philadelphia, PA
- James F. Howard, Jr., University of North Carolina, Chapel Hill, NC
- Carlayne Jackson, University of Texas Health Science Center, San Antonio, TX
- Vern Juel, Duke University, Durham, NC
- Jonathan Katz, California Pacific Medical Center, San Francisco, CA
- Jacqueline Lee, Cytokinetics, Inc., South San Francisco, CA
- Fady I. Malik, Cytokinetics, Inc., South San Francisco, CA
- Janice Massey, Duke University School of Medicine, Durham, NC
- April McVey, University of Kansas Medical Center, Kansas City, KS
- Lisa Meng, Cytokinetics, Inc., South San Francisco, CA
- Tahseen Mozaffar, University of California Irvine, Irvine, CA
- Mamatha Pasnoor, University of Kansas Medical Center, Kansas City, KS
- Jeffrey Rosenfeld, University of California San Francisco, Fresno, CA
- Donald B. Sanders, Duke University Medical Center, Durham, NC
- George Small, West Penn Allegheny Health System, Pittsburg, PA
- Yuen So, Stanford University, Stanford, CA
- Annabel K. Wang, University of California Irvine, Irvine, CA
- David Weinberg, Neurocare Center for Research, Brighton, MA
- Andrew A. Wolff, Cytokinetics Inc., South San Francisco, CA, USA

Study Coordinators:

- University of Kansas: Mimi Michaels
- University of North Carolina: Manisha Chopra
- University of California Irvine: Veronica Martin
- University of Texas Health Science Center: Pamela Kittrell
- Johns Hopkins University: Kristen Riley
- Baylor College of Medicine: Clarie MacAdam
- Duke University School of Medicine: Kate Beck
- University of California San Francisco: Christine Banda
- Drexel University College of Medicine: Christine Barr
- West Penn Allegheny Health System: Michelle Dulashaw
- Neurocare Center for Research: Renée Bell

Study Evaluators

- University of Texas Health Science Center: Deborah Myers
- Johns Hopkins University: Lora Clawson
- Drexel University College of Medicine: Sarah Feldman
- University of California San Francisco: Kimberly Voelz
- University of Kansas: Laura Herbelin
